# Benzylfentanyl as a Surrogate Template for Fentanyl-Selective Imprinted Polymers

**DOI:** 10.3390/polym15183669

**Published:** 2023-09-06

**Authors:** Md. Ragib Hasan, David A. Spivak

**Affiliations:** Department of Chemistry, Louisiana State University, Baton Rouge, LA 70803, USA; mhasa25@lsu.edu

**Keywords:** fentanyl, benzylfentanyl, molecularly imprinted polymer (MIP), narcotics, molecular recognition

## Abstract

The illicit use of fentanyl has led to hundreds of thousands of opioid-related deaths worldwide. Therefore, the detection of fentanyl by law enforcement and recreational users is of utmost importance. However, current detection methods are expensive, time-consuming, require special storage conditions, and necessitate complex instrumentation that is generally unportable and requires skilled personnel to operate. An alternative approach would be using molecularly imprinted polymers (MIPs) as the recognition component of a handheld sensor, testing strip, or color-based assay. In this work, a molecularly imprinted polymer was constructed using the functional monomer methacrylic acid (MAA) and the cross-linking monomer ethyleneglycol dimethacrylate (EGDMA), with benzylfentanyl (Bfen) as the template. The use of benzylfentanyl is advantageous because it closely mimics fentanyl’s structure but does not cause any physiological narcotic effects. Important studies herein determined the optimum ratio of the template/functional monomer, with subsequent evaluations of selectivity of the MIP for the template and fentanyl versus the commonly encountered narcotics such as methamphetamine, cocaine, and heroin. The data obtained from the HPLC analysis showed that the Bfen-MIP was successful in selectively binding the template and actual fentanyl, better than other common narcotics.

## 1. Introduction

Fentanyl is used in clinical settings to manage severe pain in cases such as cancer patients or after surgery. However, it is increasingly being used illicitly, either as a stand-alone drug or combined with other substances such as cocaine, heroin, or counterfeit prescription pills, which can result in unintentional overdoses and fatalities [1,2]. The illicit use of fentanyl has led to a surge in opioid-related deaths worldwide due in part to the fact that fentanyl is up to 100 times more potent than morphine, and just a small amount (approximately 2 mg) can be lethal. In the United States, for example, the Centers for Disease Control and Prevention (CDC) reported over 70,000 deaths from overdoses involving fentanyl in 2021. Canada has also seen a significant increase in fentanyl-related deaths, with greater than 75% of all opioid deaths post-2020 due to fentanyl [3]. While there are fewer but significant numbers of deaths in Europe and the rest of the world, the European Monitoring Centre for Drugs and Drug Addiction (EMCDDA) has put out warnings regarding rampant fentanyl diffusion in different areas of Europe as well as the presence of new formulations of fentanyl analogs, with some of them up to 10,000 times more potent than morphine [4].

Addressing the fentanyl problem requires a multifaceted approach, including strategies to reduce the availability of illicit fentanyl through law enforcement efforts, improving access to treatment for opioid addiction, and increasing public awareness of the dangers of fentanyl. Additionally, there is a need for improved detection and analytical methods to identify fentanyl for law enforcement or for the detection of fentanyl contamination in other drugs. Current methods of detection and analysis of fentanyl and its analogs involve various spectroscopic or chromatographic techniques such as LC-HRMS [5], SPME-GC-MS [6], GC-IR [7], GC-ECD [8], LC-UV [9], IMS [10], and other methods [11,12,13]. However, these methods are expensive, time-consuming, require large instrumentation that is generally unportable, and necessitate skilled individuals to carry out the test procedures. Other methods for visual and sensitive detection include surfactant-based colorimetric tests [14,15], electrochemical sensors [16,17], and immunoassays, for instance, ELISAs, EMITs, and LFAs [18]. Recently, antibody-based fentanyl test strips (FTSs) have attracted attention due to their small size, portability, and simplicity of use; however, antibodies suffer from poor stability under varying conditions, prolonged manufacture times, and can provide false-positive responses.

**Scheme 1:**
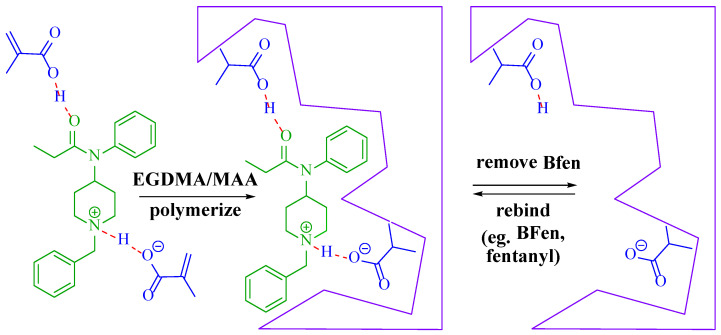
Outline of molecular imprinting using benzylfentanyl as a template where the green structure is the template, blue structures are the functional monomers and the purple outline indicates the binding cavity formed by the crosslinking and functional monomers.

The development of molecularly imprinted polymers (MIPs) as the molecular recognition component for fentanyl sensors and separations is a promising advancement in this area. MIPs are very stable and can maintain their recognition properties for decades, even when exposed to high or low temperatures, different solvents, and variable humidity. Moreover, MIPs are inexpensive and can be adjusted chemically to maximize performance characteristics. The synthesis of MIPs begins with the formation of a complex between a targeted template molecule (e.g., fentanyl) and monomer(s), which is most easily accomplished via noncovalent interactions by simply mixing these components in a solvent (Scheme 1). The complex is polymerized, and the template is removed by simple extraction to leave cavities in the polymer that are imprinted to match the shape of the template and provide functional groups in a complementary interactive array to the template. There have been two previous reports of molecular imprinting of fentanyl, the earliest describing a fentanyl-imprinted silicate xerogel developed for extraction based assays that exhibited an increase in adsorbed fentanyl versus nonimprinted xerogel materials with selectivity versus donepezil [19]. The second report imprinted carboxyl-fentanyl in nanoparticles using a multimonomer mixture applied to a long-period grating sensor that selectively detected the template [20].

To initiate a long-term goal of optimizing fentanyl-specific binding MIPs, this study explores the effect of changing the relative template ratio versus the amount of functional monomer employed. The optimum formulation was subsequently evaluated for selectivity versus narcotics that are often laced with fentanyl, such as methamphetamine, heroin, and cocaine. Because of the fatal toxicity of fentanyl, a structurally close analog, benzylfentanyl (BFen), was used for the imprinting process and binding compared to genuine fentanyl. The analog BFen only differs in structure from fentanyl by a single carbon reduction in the N-2-phenylethyl group of fentanyl and therefore was anticipated to best mimic fentanyl during imprinting. The use of an analog versus actual fentanyl will create safe MIPs without toxicity (benzylfentanyl is pharmacologically inactive) [21,22] and will eliminate the contamination of fentanyl samples by trace amounts of fentanyl remaining in the MIP causing inaccurate detection results. The outcome of this study sets the stage for further optimization studies toward improving the binding uptake and selectivity for fentanyl in MIPs, e.g., the use of different monomers, templates, crosslinkers, and monomer/component ratios.

## 2. Materials and Methods

### 2.1. Materials

Ethylene glycol dimethacrylate (EGDMA, Aldrich, St. Louis, MI, USA) and methacrylic acid (MAA, Aldrich) were distilled in vacuo over boiling chips prior to polymerization. Benzylfentanyl, acetyl-benzylfentanyl (ABF), and benzoyl-benzylfentanyl (BBF) were synthesized as described herein. Fentanyl was synthesized according to the method in reference [23]. Heroin, cocaine, methamphetamine, and 2,2-azobisisobutyronitrile (AIBN) were purchased from Sigma-Aldrich and used without further purification. The solvents used were HPLC-grade, obtained from VWR (Radnor, PA, USA), and used without further purification.

### 2.2. Synthesis of Benzylfentanyl, Acetyl-Benzylfentanyl, and Benzoyl-Benzylfentanyl

**Scheme 2:**
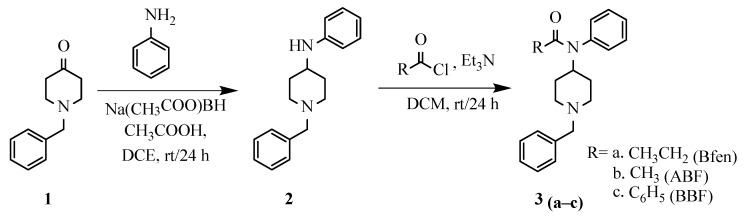
Overall strategy for the synthesis of benzylfentanyl and its derivatives.

Benzylfentanyl and its derivatives were synthesized using the route shown in Scheme 2, as described below, following similar routes found in the literature (with modifications) to obtain the highest overall yield of any publication [23,24].

#### 2.2.1. Synthesis of 1-Benzyl-4-(phenylamino) Piperidine (**2**)

In an RBF, aniline (4.92 g, 53 mmol) and 1-benzyl-4-piperidone (**1**) (4.0 g, 21 mmol) were dissolved in 50 mL of 1,2-dichloroethane, followed by the addition of AcOH (1.27 g, 21 mmol). Afterward, the mixture was reacted with sodium triacetoxyborohydride (6.72 g, 32 mmol) and stirred at RT for 24 h. Subsequently, 1 N NaOH was added to the reaction mixture to quench the remaining sodium triacetoxyborohydride, followed by the extraction of the product with 1,2-dichloroethane. The drying of the organic layer was accomplished using MgSO_4_ followed by vacuum concentration to give either a yellow oil or a solid. Purification of the product was carried out using either column chromatography utilizing EtOAc:CHCl_3_ 50:50 or recrystallization in hexane to obtain a 90% yield. ^1^H NMR (400 MHz, (CD_3_)_2_SO) δ 7.21–7.33 (m, 5H), δ 7.02 (t, 2H), δ 6.55 (d, 2H), δ 6.46 (t, 1H), δ 5.35 (d, 1H), δ 3.46 (s, 2H), δ 3.17 (quin, 1H), δ 2.75 (d, 2H), δ 2.06 (t, 2H), δ 1.85 (d, 2H), δ 1.38 (dq, 2H).

#### 2.2.2. Acylation of 1-Benzyl-4-(phenylamino) Piperidine (**2**)

Et_3_N (1.14 g, 11 mmol) was added to 1-Benzyl-4-(phenylamino) piperidine (**2**) (1.0 g, 3.8 mmol) in 15 mL of DCM. Subsequently, the appropriate acid chloride (11 mmol) was added to the solution and stirred for 24 h at RT. Upon completion of the reaction, the reaction mixture was washed with 3 × 15 mL of 1 N NaOH and 2 × 15 mL of 5 N NaOH, then extracted with DCM. The drying of the organic layer was accomplished using MgSO_4_ followed by vacuum concentration to give either a yellow oil or a solid. Purification of the product was carried out using either column chromatography utilizing EtOAc:CHCl_3_ 30:70 or recrystallization in hexane, yielding the product in the range of 70–87%. Benzylfentanyl (**3a**): ^1^H NMR (400 MHz, (CD_3_)_2_SO) δ 7.47–7.39 (m, 3H), δ 7.27–7.17 (m, 7H), δ 4.43 (quin, 1H), δ 3.37 (s, 2H), δ 2.75 (d, 2H), δ 1.97 (t, 2H), δ 1.81 (q, 2H), δ 1.65 (d, 2H), δ 1.17 (dq, 2H), δ 0.86 (t, 3H). Acetyl-benzylfentanyl (**3b**): ^1^H NMR (400 MHz, (CD_3_)_2_SO) δ 7.48–7.40 (m, 3H), δ 7.28–7.24 (m, 2H), δ 7.21–7.15 (m, 5H), δ 4.41 (quin, 1H), δ 3.37 (s, 2H), δ 2.78 (d, 2H), δ 1.97 (t, 2H), δ 1.68 (d, 2H), δ 1.60 (s, 3H), δ 1.21 (dq, 2H). Benzoyl-benzylfentanyl (**3c**): ^1^H NMR (400 MHz, (CD_3_)_2_SO) δ 7.34–7.26 (m, 5H), δ 7.22 (dd, 2H), δ 7.18–7.07 (m, 6H), δ 6.97 (dd, 2H), δ 4.80 (quin, 1H), δ 3.65 (s, 2H), δ 3.09 (d, 2H), δ 2.31 (t, 2H), δ 1.91 (d, 2H), δ 1.73 (dq, 2H).

### 2.3. Polymer Preparation

Benzylfentanyl (2.7 mmol) was dissolved in chloroform (4 mL) in a 13 mm × 100 mm screw cap tube. To this solution, 21 mmol EGDMA, 5.4 mmol MAA, and 0.54 mmol AIBN were added. The nonimprinted control polymer (NIP) was prepared similarly without the introduction of the benzylfentanyl template. Nitrogen gas was bubbled into each solution to purge for 5 min, followed by capping the tube and sealing it with parafilm.

The samples were placed into a constant-temperature water/ethylene glycol bath and equilibrated to 19 °C equipped with a standard laboratory UV light source (a Canrad–Hanovia medium-pressure mercury arc lamp, 450 W) placed in a borosilicate double-walled immersion well immersed in the bath, and the polymerization was initiated photochemically. The system’s temperature was maintained by both the constant-temperature bath and the cooling jacket surrounding the lamp. The polymerization reaction was run for 8 h; afterward, the sample tubes were manually cracked open, and the polymer was removed. The polymers were lightly crushed for Soxhlet extraction using methanol overnight before grinding and sizing.

### 2.4. Particle Sizing

MIP materials made in bulk are most often ground and then sized using a sieve before packing in columns that are subsequently evaluated for binding. Thus, the polymers were ground utilizing a mortar and pestle, and the particles were sized using USA Standard Testing Sieves (VWR, Radnor, PA, USA) or Whatman #1 filter paper. The sieved particles were collected in the 25–37-micron range; the particles passing below the 25-micron sieves were further filtered through Whatman #1 filter paper to obtain particles in the 11–25-micron range. Prior to evaluation, both particle sizes were separately slurry packed into HPLC columns (10 cm × 4.1 mm i.d.) using a Beckman 1108 Solvent Delivery Module Beckman Instruments, Palo Alto, CA, USA) and eluted overnight with MeCN/H_2_O: 95/5 (*v*/*v*), after which the baseline was steady, indicating no further BFen needed to be removed.

### 2.5. Chromatographic Tests

A Hitachi HPLC system comprising an L-7400 detector and a Hitachi L-7100 pump was employed for the HPLC analysis, which was run at room temperature (22 °C) under isocratic conditions. Samples comprising 20 µL of a 0.1 mM solution of different analytes in acetonitrile were injected into the HPLC, and the chromatographic values detected at λ = 254 nm were reported as the average value of triplicate runs. Acetone was used as the inert substance to determine the void volume. Calculation of the capacity factors was performed using the relation $\left(k^{\prime }=\frac{t_{R}-t_{V}}{t_{V}}\right)$, where $t_{R}$ is the retention time of the analyte, and $t_{V}$ is the void volume. The imprinting factor (IF) was calculated as the ratio of capacity factors from the imprinted column over the nonimprinted column ($\frac{k_{MIP}^{\prime }}{k_{NIP}^{\prime }}$). Finally, to determine the selectivity of the MIP, the cross-binding selectivity was calculated by the relation $\left(Cross-Binding\;Selectivity=\frac{k_{x}^{\prime }}{k_{Bfen}^{\prime }}\right)$, where $k_{x}^{\prime }$ is the capacity factors of the analyte and $k_{Bfen}^{\prime }$ is the capacity factors of benzylfentanyl.

## 3. Results and Discussion

### 3.1. Optimization of MIP Parameters

There are a number of variables that affect the optimization of selective binding by the BFen-MIP, such as the template/functional monomer ratio, particle size, mobile phase composition, and flow rate. Each of these parameters was evaluated in turn before the final evaluation of binding and selectivity of fentanyl, its analogs, and select narcotics often found laced with fentanyl or its derivatives.

#### 3.1.1. Optimization of Template/Functional Monomer Ratio

The first variable investigated was the optimum template/functional monomer ratio, which was carried out by the synthesis of five different BFen-MIPs incorporating different quantities of the BFen template, while at the same time keeping the formulation of functional monomer (MAA)/crosslinker (EGDMA)/initiator (AIBN) at the value 20:78:2. It should be noted that these polymer components add up to 100% of the MIP material, which does not take into account any of the templates because the template is removed in the final step of MIP preparation. Therefore, the template does not contribute to the composition of the final material. 

The ratios of template/functional monomer tested were 1:10, 1:5, 1:2.5, 1:2, and 1:1, which provided a comprehensive range of data to observe trends in MIP performance. The five synthesized MIPs were evaluated using the imprinting factor (IF) as the figure of merit, following the methods described in the “*Chromatographic Tests*” portion of the “Materials and Methods” section (*vide supra*). The IF measures the increase in the ratio of BFen binding to the imprinted polymer versus the nonimprinted polymer, in essence providing a measure of the “imprinting effect”, where better imprinting is indicated by larger IF values.

**Figure 1:**
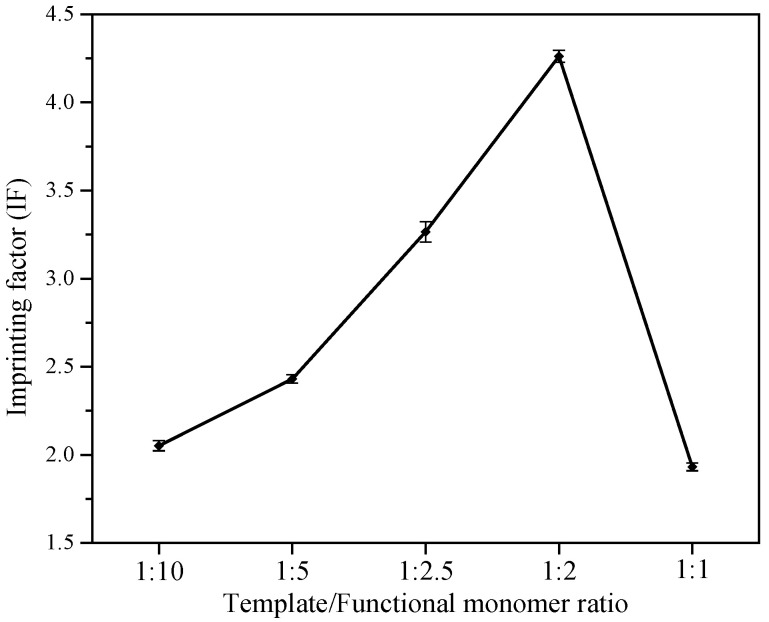
Imprinting factors (IFs) for each MIP with different template/functional monomer ratios indicating optimization at ratio 1:2.

**Scheme 3:**
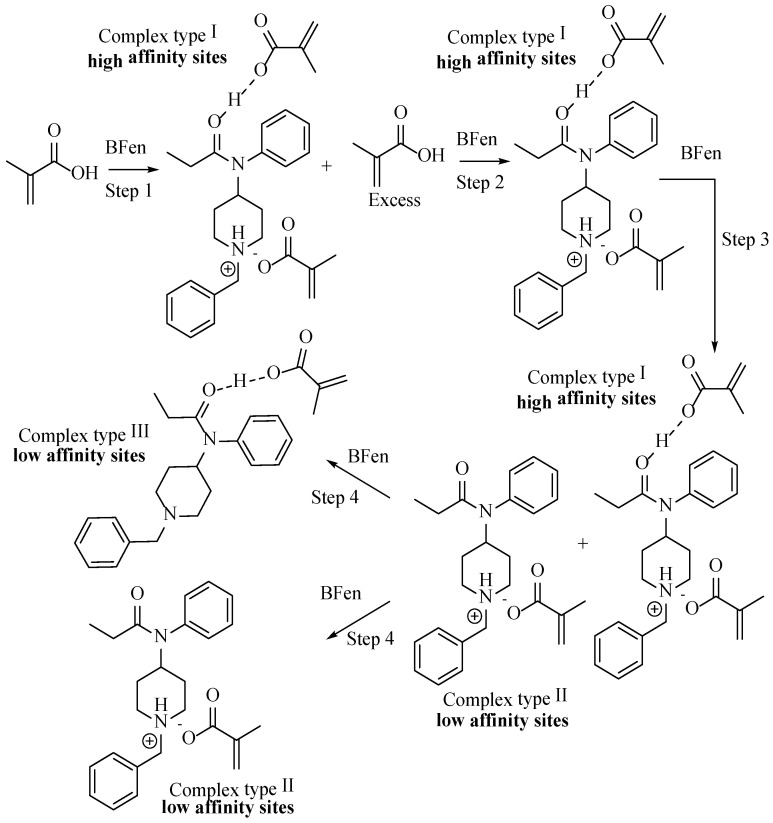
Prepolymer complexes formed as the ratio of template/functional monomer is increased; the optimal template/functional monomer ratio of 1:2 was determined to form after step 2.

The results are shown in Figure 1, indicating the best IF occurs at a template/functional monomer ratio of 1:2. This optimum ratio can be explained by the template/functional monomer complexes illustrated in Scheme 3, showing the solution phase intermediates in template–functional monomer complex formation as the amount of template is increased, which is similarly described for other MIPs [25]. After BFen is added to MAA in step 1 of Scheme 3, it is envisioned that the template is fully complexed to yield “complex I”, with the remaining excess MAA not complexed to the template. As the template concentration increases, as in the case of the 1:5, 1:2.5, and 1:2 ratios, the absolute concentration of “complex I” increases, which leads to a proportional increase in the total number of binding sites per gram of MIP, which in turn increases the IF (per gram of MIP), as shown in Figure 1. 

This means the “imprinting effect” improves, as indicated by the increased value, as the number of binding sites is increased per gram of MIP. When the ratio reaches 1:2, the maximum concentration of “complex I” is reached, and there is no longer any excess MAA. At this point, the maximum number of “high affinity” binding sites has been reached, leading to the maximum “imprinting effect” or IF observed. Further increasing the template to give a ratio of 1:1 decreases the number of “high affinity” sites (“complex I”) which have become low-affinity sites (“complex II” and “complex III”). The low-affinity sites are a result of fewer functional monomers bound to the template, lowering the enthalpic and entropic contributions to binding. Thus, the overall “imprinting effect” goes down as the high-affinity sites are substituted by the low affinity sites. From these results, the ratio 1:2 template/functional monomer was chosen for subsequent studies.

#### 3.1.2. Particle Size Optimization

There have been several reports that assessed the chromatographic performance of different-sized MIP particles, from which some generalizations can be made [26,27]. One of the clearest findings is MIP sizes greater than 63 µm show poorer selectivity and lower retention time due to mass transfer issues of solutes through the larger particles. On the other hand, there have been mixed findings for MIP particles ground to smaller sizes. Previous studies have shown that particle size ranges above 25 µm have provided better binding results than particles smaller than 25 µm, despite an increased number of theoretical plates and fewer problems anticipated with mass transfer associated with smaller particles in chromatography [26]. Therefore, for this study, two different particle size ranges were investigated; the larger-sized particles were obtained by sieving to the 25–37 µm range, while the smaller-sized particles in the 11–25 µm range were obtained by filtering the particles below 25 µm through Whatman #1 filter paper.

Two separate columns were packed with a slurry of differently sized MIPs and evaluated using a mobile phase system consisting of MeCN/H_2_O, 99.9/0.1, respectively. The results shown in Table 1 indicate that the 25–37 µm particle sizes produced better IF values compared to the particle size range 11–25 µm, which correlates well with the findings of previous studies [26,27].

A priori, it could be envisaged that the smaller particles should provide a better IF due to shorter distances of path length diffusion, resulting in quicker mass transfer kinetics of substrates, increased surface accessibility, and better access to binding sites buried internally in the particles. However, the data show that this is not the case, which has been attributed previously to a scenario whereby a considerable number of binding sites were postulated to have been destroyed during the grinding process to smaller-sized particles. Based on these findings, the 25–37 µm sized particles were chosen for further studies.

#### 3.1.3. Mobile Phase Composition and Flow Rate

The mobile phase conditions were evaluated and optimized using different proportions of water and acetonitrile, and it was first determined that the elution of BFen in MeCN/H_2_O: 95/5 (*v/v*) gave retention values close to the void volume, indicating that the mobile phase was too polar. Systematically reducing the amount of water in MeCN to 1% and subsequently to 0.1% resulted in longer elution times, with 100% MeCN providing the longest peak elution times, indicating the highest binding affinity. Mobile phases incorporating 0.1% and 1% formic acid in MeCN were also studied to determine if the protonation state of the BFen had an effect on binding; unfortunately, the retention times were reduced. Thus, the acid component provided an unacceptably high eluotropic strength, and correspondingly, it was determined that the best mobile phase was 100% MeCN. In addition, several flow rates were evaluated (0.1, 0.5, and 1.0 mL/min); however, the IF stayed the same in all cases, and the faster flow rate of 1.0 mL/min was chosen for further studies.

### 3.2. Evaluation of Selectivity

The selectivity of the MIP for the template BFen, and more importantly fentanyl, was compared to other narcotics that have been reported to be contaminated with fentanyl, such as methamphetamine, cocaine, and heroin. In addition, two analogs of fentanyl, acetyl-benzylfentanyl (ABF) and benzoyl-benzylfentanyl (BBF), with structural similarities to fentanyl, were also examined.

**Figure 2:**
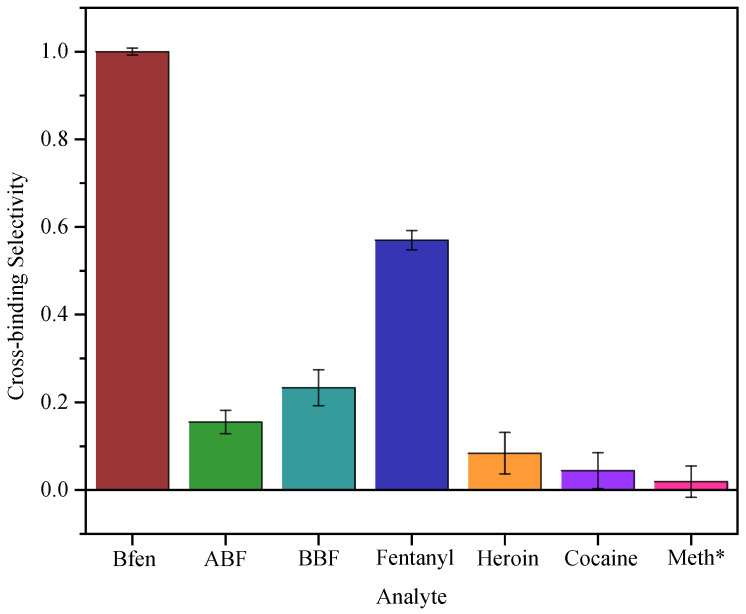
Cross-binding selectivity of the optimized BFen-MIP incorporating a template/functional monomer ratio of 1:2. Adapted from reference [29]. * Meth = methamphetamine.

**Figure 3:**
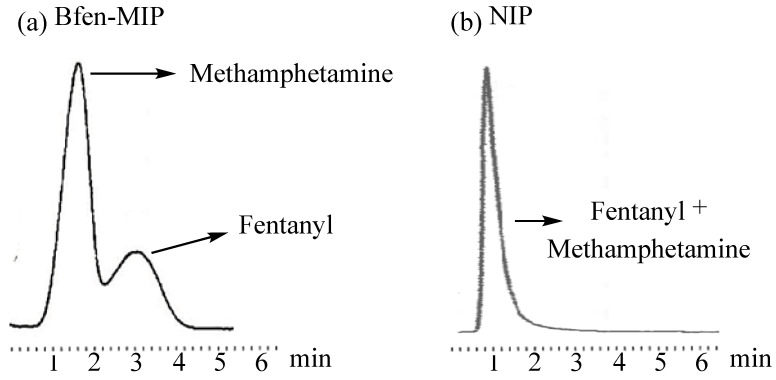
Chromatographic separation shown for fentanyl and methamphetamine on the BFen-MIP (**a**), whereas no separation was found for the same mixture on the NIP (**b**). Adapted from reference [29].

The chromatographic results from the experiments are shown in Figure 2, showing the best selectivity found for BFen, for which the MIP was originally developed. This is the usual finding for MIP technology, i.e., the template binds best to its own imprinted polymer [28]. To evaluate the cross-binding selectivity (CBS) of the other compounds tested, the capacity factors were normalized relative to BFen binding, as described in Section 2.5. The second-best recognized analyte was fentanyl, a very important finding that opens the door to using this MIP for promising applications in the detection and/or separation of fentanyl from other substances. For example, Figure 3 shows a typical MIP-based separation of fentanyl from methamphetamine, demonstrating the ability of the BFen-MIP to distinguish fentanyl laced in another narcotic. This suggests that the BFen-MIP could serve as the recognition component of fentanyl sensors for law enforcement or in commercial test strips for point-of-care analysis. The related compounds ABF and BBF also appeared to show significantly less binding in spite of their structural similarity to fentanyl. The higher binding seen for BBF versus ABF is possibly due to increased nonspecific binding that may occur due to the presence of the more hydrophobic benzene group.

The second-best recognized analyte was fentanyl, a very important finding that opens the door to using this MIP for promising applications in the detection and/or separation of fentanyl from other substances. For example, Figure 3 shows a typical MIP-based separation of fentanyl from methamphetamine, demonstrating the ability of the BFen-MIP to distinguish fentanyl present in another narcotic. This suggests that the BFen-MIP could serve as the recognition component of fentanyl sensors for law enforcement or in commercial test strips for point-of-care analysis. The related compounds ABF and BBF also appeared to show significantly less binding in spite of their structural similarity to fentanyl. The increased binding seen for BBF versus ABF is possibly a result of increased nonspecific binding to the substantially more hydrophobic benzene group.

**Figure 4:**
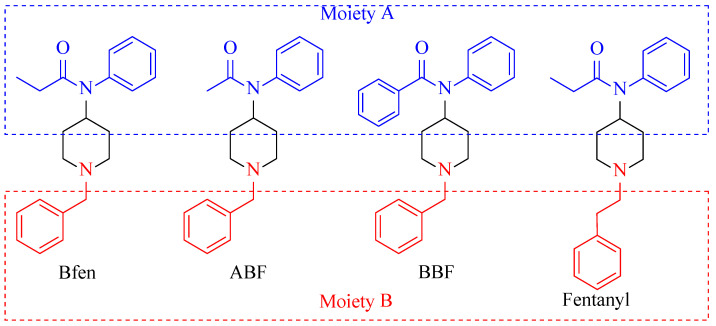
Differential locations controlling binding of fentanyl and fentanyl analogs by the BFen-MIP.

The significant difference in binding of BFen and fentanyl versus ABF and BBF is curious, and points to the substructures of fentanyl and its analogs having greater influence on binding. For instance, Figure 4 splits BFen into two moieties representing the top and bottom halves of the molecule. The top moiety (A) was found in both BFen and fentanyl, which were the highest binding analogs. However, ABF and BBF had changes in moiety A and suffered a severe loss of binding. On the other hand, ABF and BBF are most similar to BFen with respect to moiety B versus fentanyl, yet fentanyl showed better binding to the MIP. This suggests the binding of fentanyl analogs is controlled more by moiety A than moiety B. Nonetheless, moiety B does influence binding, as seen in the approximately 40% lowering of binding by fentanyl versus BFen, which only differed by one methylene unit in moiety B. This unexpected finding could point toward the design of future analogs that could elicit a higher affinity for fentanyl. Methamphetamine, cocaine, and heroin essentially showed negligible binding affinity to the imprinted polymer, as shown by elution as expected, virtually at the void volume.

## 4. Conclusions

A fentanyl-selective MIP was synthesized using BFen as the template, which is anticipated to be the analog most closely resembling fentanyl. A tremendous advantage of using BFen is its significantly lower toxicity compared to fentanyl, as well as the important benefit of using analogs as the MIP template, which eliminates any interference in detection by leaching any remaining template. Although the literature has two examples of fentanyl-binding MIPs, neither of these reports discussed the development of MIP matrix formulation to maximize the binding and/or selectivity of fentanyl. With the long-term goal of optimizing fentanyl-specific binding MIPs, a traditional polymer formulation utilizing EGDMA as the crosslinker and MAA as the functional monomer was chosen as the first generation of BFen-MIPs for study. This formulation was successful in selectively binding the template and genuine fentanyl. The best selectivity was found for BFen, which is not surprising since most MIP studies report that the template used for imprinting turns out to be the strongest-binding analyte. However, it was very encouraging that fentanyl exhibited the next best selectivity in binding to the MIP. The two other benzylfentanyl analogs tested also showed binding to the MIP to a lesser degree but significantly better than the nonrelated narcotics in comparison. In general, for MIPs, compounds that are most similar in structure to the template tend to exhibit better binding compared to nonrelated compounds. It is anticipated that this phenomenon could be useful for the detection of a large family of fentanyl analogs which are often encountered as contraband. The low binding of nonrelated compounds such as heroin, cocaine, and methamphetamine could be strategically employed in monitoring contamination with fentanyl analogs. Improvements in the fentanyl binding and selectivity using different monomers and crosslinkers, as well as optimization of the MIP component ratios, are currently under investigation. These materials will be further incorporated into detection formats and assays that will someday save many lives.

## Figures and Tables

**Table 1:** Imprinting factors for different-sized MIP particles.

Entry #	Particle Size (µm)	IF
1	25–37	4.26 (±0.03)
2	11–24	2.48 (±0.04)

## Data Availability

Data are available from the corresponding author upon request.
